# Barriers to sEMG Assessment During Overground Robot-Assisted Gait Training in Subacute Stroke Patients

**DOI:** 10.3389/fneur.2020.564067

**Published:** 2020-10-19

**Authors:** Michela Goffredo, Francesco Infarinato, Sanaz Pournajaf, Paola Romano, Marco Ottaviani, Leonardo Pellicciari, Daniele Galafate, Debora Gabbani, Annalisa Gison, Marco Franceschini

**Affiliations:** ^1^Department of Neurological and Rehabilitation Sciences, IRCCS San Raffaele Pisana, Rome, Italy; ^2^Department of Human Sciences and Promotion of the Quality of Life, San Raffaele University, Rome, Italy

**Keywords:** surface electromyography, overground robot-assisted gait rehabilitation, stroke, clinical applications, sEMG barriers

## Abstract

**Background:** The limitation to the use of ElectroMyoGraphy (sEMG) in rehabilitation services is in contrast with its potential diagnostic capacity for rational planning and monitoring of the rehabilitation treatments, especially the overground Robot-Assisted Gait Training (o-RAGT).

**Objective:** To assess the barriers to the implementation of a sEMG-based assessment protocol in a clinical context for evaluating the effects of o-RAGT in subacute stroke patients.

**Methods:** An observational study was conducted in a rehabilitation hospital. The primary outcome was the success rate of the implementation of the sEMG-based assessment. The number of dropouts and the motivations have been registered. A detailed report on difficulties in implementing the sEMG protocol has been edited for each patient. The educational level and the working status of the staff have been registered. Each member of staff completed a brief survey indicating their level of knowledge of sEMG, using a five-point Likert scale.

**Results:** The sEMG protocol was carried out by a multidisciplinary team composed of Physical Therapists (PTs) and Biomedical Engineers (BEs). Indeed, the educational level and the expertise of the members of staff influenced the fulfillment of the implementation of the study. The PTs involved in the study did not receive any formal education on sEMG during their course of study. The low success rate (22.7%) of the protocol was caused by several factors which could be grouped in: patient-related barriers; cultural barriers; technical barriers; and administrative barriers.

**Conclusions:** Since a series of barriers limited the use of sEMG in the clinical rehabilitative environment, concrete actions are needed for disseminating sEMG in rehabilitation services. The sEMG assessment should be included in health systems regulations and specific education should be part of the rehabilitation professionals' curriculum.

**Clinical Trial Registration:** www.ClinicalTrials.gov, identifier: NCT03395717.

## Introduction

In neurorehabilitation services, new devices have been widely used for both assessment and rehabilitation. The technology-based assessment includes instrumentation for medical imaging, for measuring electrophysiological signals (Electroencephalography, EEG; surface ElectroMyoGraphy, sEMG) and biomechanics (motion capture, inertial sensors). The technology for rehabilitation includes a wide range of devices such as: electrical stimulators, mechanical vibrators, robotics, virtual reality-based systems, etc. While the diffusion of technology for rehabilitation has been simplified by the great clinical interest in maximizing the outcomes of the rehabilitative treatment, the technology for assessing electrophysiological signals (EEG and sEMG) has been usually confined to research studies. As a consequence, the clinical acceptance of measuring tools, like sEMG, is low and such devices are not included in the usual clinical practice (1). On the other hand, devices like robots for rehabilitation have been rapidly accepted and integrated into rehabilitation services since they have been considered by clinicians as devices which could help to maximize patient recovery (2). Recent cutting-edge robots include the Wearable Powered Exoskeletons (WPEs) which allow a person to walk on hard and flat surfaces by moving the lower limbs with a pre-programmed physiological gait pattern (3–6). The success of WPEs in neurorehabilitation services is mainly due to their capacity to allow overground ambulation even in subjects who are not able to maintain the upright position (7–10), inducing a coordinated, multisensory motor control stimulation. Since these mechanisms are crucial for the restoration of motor control, the o-RAGT could be considered as a rehabilitation treatment which generates a more complex, controlled multisensory stimulation of the patient and which may modify the plasticity of neural connections through the experience of movement (11). While the literature on overground Robot–Assisted Gait Training (o-RAGT) with a WPE is rapidly growing up (12–18), studies which included technology-based assessment, like the sEMG, are restricted (19–23).

The limitation to the use of sEMG in rehabilitation services is in contrast with its potential diagnostic capacity for rational planning and monitoring of the rehabilitation treatments (24–27). A number of barriers limiting the clinical diffusion of sEMG have been recently highlighted by Feldner et al. (28). Cultural, technical and administrative barriers seem to be the principal limitation to sEMG in the clinical practice. The cultural barriers impact on the clinical acceptance of sEMG among Physical Therapists (PTs) and clinicians. In fact, low acceptance is mainly due to the insufficient knowledge to interpret the sEMG outcomes and thus to recognize the clinical relevance of sEMG. Technical barriers are often caused by the limited user confidence of some equipment which may make the acquisition and processing phases problematic. Administrative barriers include device costs and the time required to perform acquisition and processing, which are characteristics common to most biomedical technologies. The cultural and technical barriers mainly depend on the educational background of the clinical staff. Indeed, wide differences between countries exist, since the training programs of PTs and clinicians are very different, especially in approaching new technologies (29, 30). Moreover, to our best knowledge, studies on the barriers to sEMG in clinical practice lack. For these reasons, this paper focuses on introducing the sEMG in a clinical context where technology-based rehabilitation is promoted.

The aim of this work is to assess the barriers faced during the implementation of a sEMG protocol in a clinical study on o-RAGT in subacute stroke patients. The analysis of difficulties encountered during the protocol will allow us to discuss strategies and options which could help to reduce them.

## Materials and Methods

An observational study was conducted in a rehabilitation hospital (IRCCS San Raffaele Pisana of Rome) in order to investigate the barriers in implementing a sEMG-based assessment protocol in subacute stroke patients who underwent o-RAGT.

### Barriers Assessment

The primary outcome was the success rate of the implementation of the sEMG-based assessment, expressed as the percentage of successfully assessed patients relative to the total number of recruited ones. The number of dropouts and the motivations were registered. A detailed report on difficulties in implementing the sEMG protocol was edited for each patient.

The educational level and the working status of the staff involved in each phase of the study (patient recruitment; clinical assessment; sEMG acquisition; o-RAGT; sEMG analysis) were registered.

Each member of staff completed a brief survey indicating their level of knowledge of sEMG, using a five-point Likert scale: very poor (no knowledge about sEMG); poor (basic knowledge about muscle electrophysiology); fair (good knowledge of muscle electrophysiology and basic knowledge of detection/interpretation techniques); good (good knowledge of detection/interpretation techniques and ability to recognize artifacts, interference); excellent (good ability to detect, collect, process and interpret the signals).

### The Clinical Study

A pilot clinical trial was carried out on a group of subacute stroke subjects who underwent o-RAGT with a WPE. This study was a subgroup analysis from a large multicenter clinical trial assessing the effects of o-RAGT (15, 16). The inclusion and exclusion criteria, the rehabilitation protocol, and the clinical assessment procedure were provided in the previous papers of the authors (15, 16). Ethical approval of the treatment and of the evaluation protocol was granted by the Ethics Committee of IRCCS San Raffaele Pisana of Rome (date: 18/11/2015; code number: 09/15). The study protocol was registered on ClinicalTrials.gov by the unique identifier number: NCT03395717, and all subjects gave informed written consent in accordance with the Declaration of Helsinki.

### The sEMG Procedure

The subjects were screened at the beginning (T1) and at the end (T2: three weeks after T1) of the o-RAGT with both clinical measures and sEMG-based assessment. The sEMG-based assessment was carried out for assessing electromyographic activity during the following walking trials: at T1 and T2 during ecological overground gait at a self-selected speed along a 10-m walkway with assistance (e.g., crutches with or without antebrachial support, walker, tripod stick, ankle support orthoses, etc.); and during the first session of o-RAGT (o-RAGT_1_). Two gait tasks for each walking trial were collected. The total duration of the experimental session was about 2 h. An eight-channel wireless sEMG device (FREEEMG 1000—BTS Bioengineering, Milan, Italy) was used to acquire (sampled at 1 kHz, filtered at 8–500 Hz) the activity of the agonist/antagonist muscles of the distal and proximal compartment of lower limbs. The skin was abraded and cleaned with alcohol, then electrodes were placed on Tibialis Anterior (TA), Gastrocnemius Medialis (GM), Rectus Femoris (RF), and Biceps Femoris caput longus (BF) muscles of each leg according to the SENIAM guidelines (31). In order to establish the gait phases, kinematics data were measured using two electrogoniometers for knee joint measurements (BTS Bioengineering, Milan, Italy) and an inertial measurement unit (G-Sensor—BTS Bioengineering, Milan, Italy), placed on the spinous process of the fifth lumbar vertebra. The SMART Analyzer software (BTS Bioengineering, Milan, Italy) was employed for the synchronization of kinematic and sEMG signals for data pre-processing and exporting. Specifically, data from the inertial sensor were analyzed in comparison with electrogoniometers with the SMART Analyzer software, and the heel strike and toe-off gait cycle events were identified for all walking trials. The gait cycle was considered as the interval of time between heel-strikes of the same foot (i.e., the right foot). These temporal events were used for all subsequent sEMG analyses. The sEMG and temporal events were exported for further custom analysis in MATLAB (MATLAB R2019a, The MathWorks Inc., Natick, MA, USA).

The sEMG data were high-pass filtered (20 Hz, 6th-order Butterworth filter, bidirectional) and full-wave rectified. For standardization, the sEMG data were normalized to 100% of a gait cycle based on the temporal events (heel strikes and toe-offs) extracted from the kinematic data. The sEMG signals of each gait cycle were processed as follows: (1) the sEMG envelope was obtained using a moving average filter (window duration equal to 120 ms) and normalized at the maximum sEMG amplitude level; (2) the activation threshold identifying onset and offset status of muscle activity was detected as the 20% of minimum-maximum amplitude level distance (32), when kept for at least 50 ms. Subsequently, the following sEMG outcomes were extracted, considering five gait cycles: (i) the Bilateral Symmetry (BS) coefficient (33); (ii) the Co-Contraction (CC) coefficient (34); (iii) and the Root Mean Square (RMS) value (35). Details on the calculation of BS and CC coefficients are described in the Appendix.

Descriptive statistics were computed in order to appropriately explain the clinical and demographic characteristics of the sample. Data were represented for each recruited stroke subject. The sEMG outcomes were averaged from the two gait tasks for each walking trial and were used for subsequent analyses. The one-way ANalysis Of VAriance (ANOVA) was applied between T1 and T2 in order to test the treatment effect of o-RAGT on sEMG outcomes.

## Results

The study was carried out by a multidisciplinary team composed of two Medical Doctors (MDs), four Physical Therapists (PTs), and two Biomedical Engineers (BEs). The MDs were Physical Medicine & Rehabilitation specialists. The sEMG acquisition and analysis were conducted by PTs and BEs. Table 1 depicts the characteristics of the members of staff who were involved in the study. PT1 and PT2 are the PTs who worked in the clinical department and administered the o-RAGT: they hold an M.Sc. in physical therapy and the patent to use the WPE in rehabilitation. The patient recruitment and clinical assessment were conducted by PT3 and PT4, both working in the research department and having an M.Sc. and Ph.D. educational level, respectively. The sEMG acquisition phase was executed by members of the research department only (two PTs and two BEs). BE1 and BE2 were in charge of the sEMG analysis and have an M.Sc. and Ph.D. educational level, respectively. The self-administered evaluation of the sEMG knowledge shows that the PTs were unfamiliar with sEMG assessment, while BEs considered themselves as experts.

**Table 1:** Characteristics of the staff who conducted the study.

**ID**		**PT1**	**PT2**	**PT3**	**PT4**	**BE1**	**BE2**
	Education level	PT M.Sc.	PT M.Sc.	PT M.Sc.	PT Ph.D.	BE Ph.D.	BE M.Sc.
	Staff	Clinical	Clinical	Research	Research	Research	Research
	sEMG knowledge^*^	Very poor	Very poor	Fair	Very poor	Excellent	Excellent
Phased of the study	Patient recruitment			X	X		
	Clinical assessment			X	X		
	sEMG acquisition			X	X	X	X
	o-RAGT	X	X				
	sEMG analysis					X	X

*The “X” shows the phases that each member of staff was involved in*.

*PT, Physical Therapist; BE, Biomedical Engineer; M.Sc., Master of Science; Ph.D., Philosophiae Doctor*.

* *sEMG knowledge assessed by a five-points Likert scale: very poor (no knowledge about sEMG); poor (basic knowledge about muscle electrophysiology); fair (good knowledge of muscle electrophysiology and basic knowledge of detection/interpretation techniques); good (good knowledge of detection/interpretation techniques and ability to recognize artifacts, interference); excellent (good ability to detect, collect, process and interpret the signals)*.

Indeed, the educational level and the expertise of the members of staff influenced the accomplishment of the implementation of the study. The PTs involved in the study did not receive any formal education on sEMG during their course of study. Two of them (PT3 and PT4) were part of the research staff and studied the sEMG recording procedures (electrodes' positioning, basic use of the device for the sEMG acquisition) as a self-taught. However, no PTs had enough knowledge to recognize artifacts and interferences or process the signals. The BEs, on the other hand, were responsible for the entire sEMG procedure from signal acquisition to processing. The limitation of sufficient knowledge in sEMG among PTs was one of the reasons for the limited diffusion of electromyography in the rehabilitation hospital, and specifically in the clinical study on o-RAGT.

**Figure 1:**
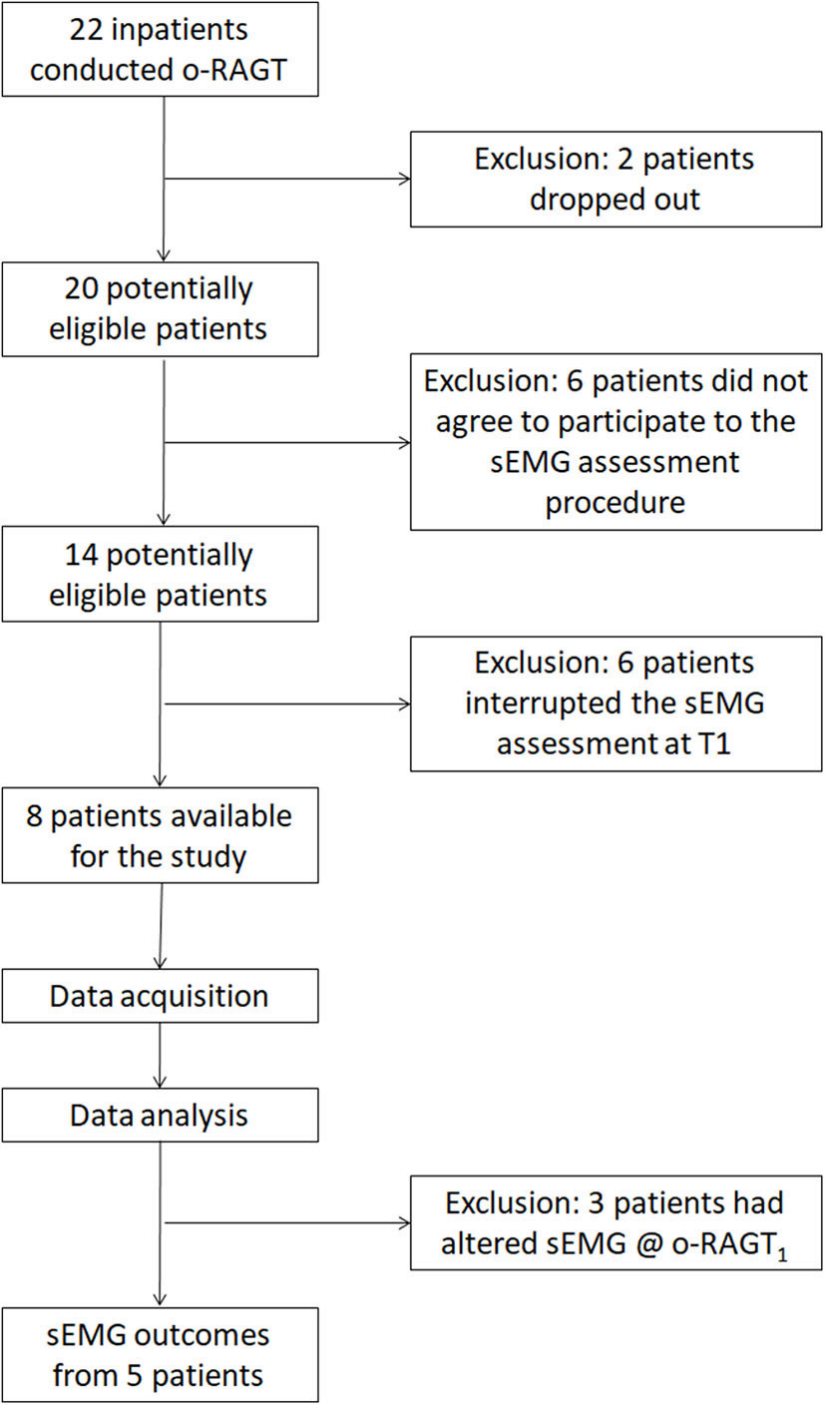
Flowchart of the experimental procedure.

A total of 22 subacute stroke patients were recruited in the clinical study. Two patients dropped out due to medical issues not related to the training or to the assessment. The remaining 20 participants completed the o-RAGT without reporting any adverse event. Fourteen patients agreed to participate in the sEMG-based assessment procedure. However, six of them could not complete the 10-m-long ecological overground gait at T1 and therefore were excluded from the study. Therefore, we recorded sEMG during ecological overground gait at T1 and T2, and at o-RAGT_1_ of a sample composed of 8 patients (the demographic and clinical characteristics of each subject are depicted in Table 2). Data acquired during the o-RAGT_1_ were partially altered and it was not possible to reliably study the muscle activity. Thus, the sEMG outcomes from 3 patients (PAT03, PAT11, PAT14) were not available. Specifically, the experimental setup for data acquisition during o-RAGT_1_ was partially influenced by the presence of the WPE: the electrode application procedure did not always comply with the SENIAM guidelines (31), because of the cumbersome WPE braces and straps. Moreover, during movement, the placement of the electrodes was moderately affected by the relative movement between the subject and the WPE. In conclusion, the number of successfully assessed patients was 5 out of the 22 initially recruited ones, and the success rate of the implementation of the sEMG-based assessment was equal to 22.7%. Figure 1 shows the flowchart of the experimental procedure.

**Table 2:** Demographic and clinical characteristics of the sample.

**ID**	**Age (years)**	**Gender**	**Affected side**	**Acute event onset time (days)**	**MAS-AL**		**MI-AL**		**FAC**		**TCT**		**10MWT (m/s)**	
					**T1**	**T2**	**T1**	**T2**	**T1**	**T2**	**T1**	**T2**	**T1**	**T2**
PAT02	64	M	R	11	3.0	3.5	60	76	2	4	87	100	0.44	0.62
PAT03	69	M	L	13	0.0	0.0	64	76	1	3	74	100	0.34	0.48
PAT06	54	F	L	29	1.0	0.0	48	65	1	3	61	100	0.16	0.32
PAT09	50	M	R	23	2.0	3.0	43	76	1	4	74	100	0.91	0.67
PAT11	76	F	R	30	1.0	1.0	64	76	2	4	87	100	0.45	0.56
PAT14	44	F	R	26	2.0	2.0	53	76	2	4	74	100	0.38	0.53
PAT18	66	M	R	12	0.0	0.0	76	82	4	5	100	100	0.44	1.41
PAT20	66	M	R	75	2.0	1.0	70	100	3	4	100	100	1.09	1.38

*M, Male; F, Female; R, Right; L, Left; MAS-AL, Modified Ashworth Scale Affected lower Limb; MI-AL, Motricity Index Affected lower Limb; FAC, Functional Ambulation Classification; TCT, Trunk Control Test; 10MWT, 10-Meter Walking Test*.

### sEMG Outcomes

**Figure 2:**
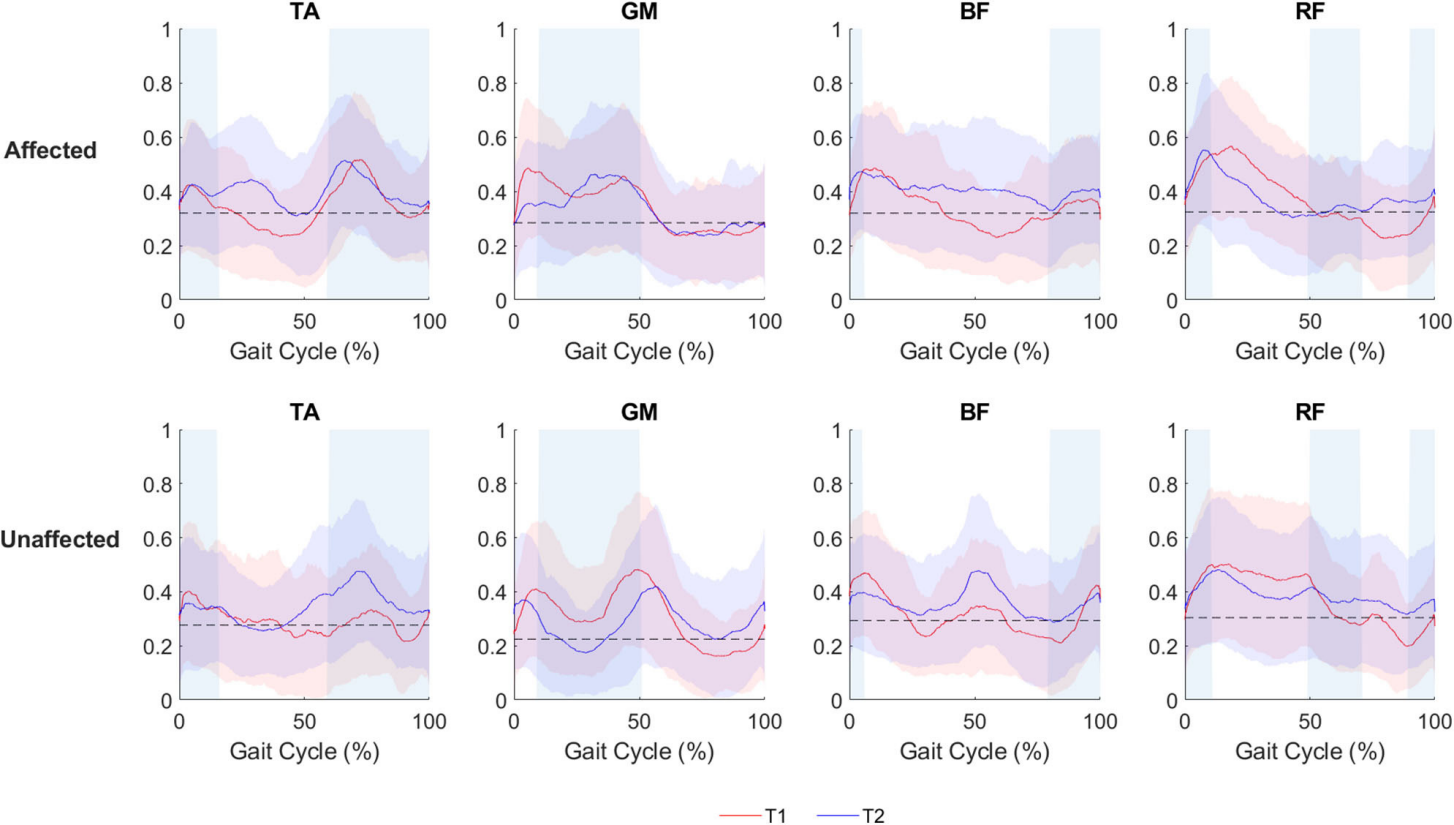
sEMG activation of the affected and the unaffected limb for all patients (*N* = 8), depicted as mean and standard deviation plot, during ecological overground gait. The red line (mean) and the red band (standard deviation) represent the sEMG envelopes (normalized with respect to the maximum sEMG amplitude level of each side) before o-RAGT (T1). The blue line (mean) and the blue band (standard deviation) represent the sEMG envelopes at the end of o-RAGT (T2). For each subject, five gait cycles have been considered. Shaded rectangular areas indicate when a muscle is active based on normative healthy adult gait, Perry and Burnfield (36).

The visual assessment of sEMG patterns during walking reveals a vast heterogeneity of the data. The mean sEMG data (Figure 2) shows volitional muscle activations during gait at both T1 and T2 during ecological overground gait. Although such muscular activity is visible on both the affected and unaffected limbs, the level of activations are characterized by variations in amplitude and timing and do not consistently correlate with the activation timing of healthy gait (36).

A traditional amplitude analysis was conducted on the sEMG data, and the following sEMG outcomes were calculated: (i) the BS coefficient; (ii) the CC coefficient; (iii) and the RMS value (35). The results of the one-way ANOVA between T1 and T2 did not reveal any significant difference. The BS coefficient registered a relevant improvement between T1 and o-RAGT_1_ in a subset of patients (PAT02 at TA and GM; PAT06 at TA, GM, BF, RF; PAT09 at TA, GM, BF; PAT11 at TA; and PAT18 at TA and BF). The effects of the 15 sessions of o-RAGT improved the BS in TA (PAT02, PAT06, PAT09, PAT11, PAT20) and moderately in RF (PAT06, PAT20). The proximal muscles registered a decrease in CC between T1 and o-RAGT_1_ (PAT02, PAT06, PAT18, PAT20) and between T1 and T2 (PAT02, PAT11, PAT14, PAT18), while the distal muscles did not. In the future, an advanced analysis of sEMG (by means of a time-varying multi-muscle coactivation function) could help to investigate the o-RAGT effects, in terms of simultaneous coactivation of a group of muscles during gait (37–39). The RMS revealed differences between the same muscles of different limbs, although the one-way ANOVA did not reveal statistical significance. Data showed a mean increase of muscle activity at T2 of TA (affected and unaffected sides), GM (affected side), BF (affected and unaffected sides), and RF (unaffected sides), although the standard deviations were high. The o-RAGT_1_ registered an increase in the RMS of TA, GM, BF, and RF of the affected side.

## Discussion

This paper focuses primarily on the difficulties encountered in the investigation of the o-RAGT technique by mean of sEMG, and secondarily on the results obtained in the o-RAGT study. This experience evidences a number of barriers limiting the implementation of the study on the effects of o-RAGT in terms of muscle activation. The low success rate (22.7%) of the protocol has been caused by several factors which can be grouped in: patient-related barriers; cultural barriers; technical barriers; and administrative barriers.

Patient-related barriers are mainly caused by low patient compliance. Except for two drop-outs caused by medical issues [no adverse events were evidenced during the o-RAGT as in (15, 16, 22)], from the 22 recruited subjects, 12 patients were excluded from the study for these reasons: (1) six patients did not agree to participate in the sEMG assessment due to tiredness; (2) six patients found the gait motor task at baseline too challenging and could not complete the sEMG assessment at T1. Both motivations depended on the subacute and complex phase of the disease. The acceptance of the sEMG assessment from patients could be improved introducing new strategies for increasing the perceived usefulness of sEMG (e.g., dedicating time to give the patient accurate and detailed information about the benefits of sEMG). Indeed, the typical impairment of stroke patients in the subacute phase should be considered in the definition of future sEMG-based protocols, and thus the analysis of basic motor tasks should be preferred.

Cultural barriers depend on the insufficient knowledge of sEMG among PTs. The role of PTs in the study was mainly as assistants of BEs during the sEMG acquisition phase. Specifically, PT3 and PT4 placed the electrodes and helped the patients to conduct the requested motor tasks. This situation is representative of the condition of PTs with respect to sEMG in Italy. In fact, the Italian academic curriculum in Physical Therapy does not include any courses on electromyography, and thus the professional figure of PT is usually focused on conventional therapy. Indeed, considering the dramatic introduction of new technologies in rehabilitation services, the role of PT should be drastically changed, and this change should start from the university curricula (40). While the bachelor's degree in PT could remain more oriented to the classical clinical role of PT, the master's degree in PT should include more technical courses. Specifically, academic courses on sEMG, EEG, gait analysis, and statistics should be added to the Italian Universities. Considering the sEMG, the PTs should be able to carry out the sEMG recording procedure (electrodes' positioning, sEMG acquisition) autonomously, using a commercial device. Moreover, the PTs should have enough knowledge to distinguish the “quality” of the acquired signals and to use commercial and user-friendly software for the basic sEMG analysis. Of course, it does not mean that the figure of BEs is not necessary for the rehabilitation hospital, but their role should be more oriented to the use of innovative prototypal sEMG devices and to the development of novel advanced signal processing techniques.

Technical barriers have been encountered during o-RAGT_1_. The sEMG signal was affected by the change of electrode location (41, 42), due to the presence of the WPE: the space between the limbs and the WPE did not allow to place the electrodes appropriately and the electrodes were partially moved by the exoskeleton during the movement of the patient. Thus, the quality of the sEMG signals of 4 patients during the o-RAGT_1_ was altered and the sEMG outcomes were unreliable. This barrier could be overcome in future studies by using high-density sEMG (43, 44) or making structural changes to the WPE.

Administrative barriers related to management and time-related issues had a negative impact on the dissemination of sEMG assessment in our clinical environment: a limited amount of time was available for the sEMG acquisition, because of the intensive schedule of rehabilitative treatments. These time-related barriers have been highlighted also by Feldner et al. (28) and by Swank et al. (21). A solution for increasing the diffusion of electromyography could be the inclusion of sEMG acquisition in the routine clinical practice for patient assessment (recognized by regional regulations for public health), thus dedicating a timeslot of the planned schedule to this procedure. Indeed, in this case, the clinical PTs should have an appropriate educational background on sEMG. Our results evidence that a multidisciplinary team is required to conduct the study because of the heterogeneity in technical skills: while the BEs, which were involved in both the sEMG acquisition and analysis, had a solid knowledge of sEMG, mainly learned during their course of study in higher education (M.Sc. and Ph.D.), the PTs did not receive any specific training on the topic during their course of study. In this context, a higher diffusion of a sEMG in an intensive rehabilitation hospital could be facilitated by the introduction of a specific education of PTs on sEMG. In our experience, while the background of clinical PTs on WPE is influenced by the need to have a patent to use the device in rehabilitation, the knowledge of sEMG is rarely supported by the Italian PT university curricula and by the need to use this technology in daily clinical practice. A solution to overcome the educational barriers could be the constitution of appropriate training of PTs on both the theory and the technical aspects of sEMG. An alternative solution could be the introduction of a new figure (i.e., the Clinical Technologist) as a healthcare professional who has the expertise to translate medical technology use into improved patient-specific procedures (45). However, in our opinion, the competence of rehabilitation professionals in the use of new technologies (i.e., sEMG) should be increased with the diffusion of specific training courses [like the one described by De la Fuente et al. (46)].

## Conclusion

In conclusion, this paper offers an insight into the barriers limiting the use of sEMG during o-RAGT in subacute stroke patients. Certainly, since a series of barriers limited the application of sEMG in the clinical rehabilitative environment, concrete actions are needed for disseminating sEMG in rehabilitation services. The sEMG assessment should be included in health systems regulations and specific education should be part of the rehabilitation professionals' curriculum.

## Data Availability Statement

The raw data supporting the conclusions of this article will be made available by the authors, without undue reservation.

## Ethics Statement

The studies involving human participants were reviewed and approved by IRCCS San Raffaele Pisana, Via della Pisana, 235, 00163 Rome, Italy. The patients/participants provided their written informed consent to participate in this study.

## Author Contributions

MG, FI, and MF have made substantial contributions to conception and design. SP, DGal, and DGab participated in the enrollment phase and carried out the o-RAGT treatment. SP, MG, and PR carried out the clinical and sEMG assessments. MO and PR designed the algorithm for sEMG data analysis. LP, PR, MO, and AG participated in the study design and coordination, and statistical analysis. SP and FI participated in the manuscript revisions. MF and FI gave the final approval of the version. All authors contributed to the article and approved the submitted version.

## Conflict of Interest

The authors declare that the research was conducted in the absence of any commercial or financial relationships that could be construed as a potential conflict of interest.
