# Supplementary material for: Barriers to sEMG Assessment During Overground Robot-Assisted Gait Training in Subacute Stroke Patients
Source: Front Neurol. 2020 Oct 19;11:564067. doi: 10.3389/fneur.2020.564067 (PMC7604287; doi:10.3389/fneur.2020.564067)
Supplement: Supplementary file 1 [file Table_1.DOCX]

Supplementary Material

# Appendix on sEMG data processing

The BS coefficient for each couple of homologous muscles is an assessment of similarity in muscle behavior between the affected and unaffected sides. It is calculated as the normalized cross-correlation function with a zero-time lag between stride envelopes [47], by applying the following formula to two *x* and *y* finite and real series:

$\hat{R}_{\mathrm{xy}}(m)=\frac{R_{xy (m)}}{\sqrt{R_{\mathrm{xx}}\left( 0 \right) R_{\mathrm{yy}}\left( 0 \right)}}$ , for m=0 with

$$R_{\mathrm{xy}}(m)=\left\{ \begin{aligned} \sum_{n=0}^{N-m-1} x_{n+m}y_{n}, m\geq0, \\ R_{\mathrm{xy}}\left( -m \right), m<0 \end{aligned} \right.$$

A value $\hat{R}_{\mathrm{xy}}\left( 0 \right)$near 0 or near 1 indicates a low or high similarity between the two sides (affected and unaffected) muscle activity respectively.

The CC coefficient is representative of the agonist-antagonist muscle couple coactivation. The stride sub-intervals above the threshold identifying on-off muscle status were employed to calculate the CC coefficient: overlapping sub-intervals for a duration higher than 30 ms between the couple of agonist-antagonist muscles contributed to measure the co-activation time as a percentage of the stride cycle.
